# Cognitive Ability Testing in the Workplace: Modern Approaches and Methods

**DOI:** 10.3390/jintelligence13060068

**Published:** 2025-06-11

**Authors:** Anne E. Kato, Yuliya M. Cheban-Gore, Charles A. Scherbaum, Harold W. Goldstein

**Affiliations:** 1 School of Business, Government, and Economics, Seattle Pacific University, Seattle, WA 98119, USA; 2 American Institutes for Research, Arlington, VA 22202, USA; yuliya.cheban@gmail.com; 3 Baruch College, City University of New York, New York, NY 10010, USA; charles.scherbaum@baruch.cuny.edu (C.A.S.); harold.goldstein@baruch.cuny.edu (H.W.G.)

## 1. Introduction

Despite the increasing importance of cognitive abilities in today’s workplaces and growing dissatisfaction with the status quo of cognitive ability assessment, the ways that cognitive abilities are conceptualized and measured in the workplace have changed very little over the past century (Scherbaum et al. 2012). In contrast, other fields such as clinical and cognitive psychology, developmental and educational research, and neuroscience have made considerable advances in understanding cognitive ability constructs, their role in the modern world, and how they can be measured (Goldstein et al. 2009; Scherbaum and Goldstein 2015). However, these advances have had a minimal influence on the conceptualization and measurement of cognitive abilities in the workplace. This gap underscores the need for continued research that draws on the developments in other fields and equips organizations with more effective, modern approaches to assessing cognitive abilities. 

This Special Issue presents seven articles that showcase innovative research grounded in modern theories of cognitive ability, modern analytical approaches, and novel measurement methods. Together, these contributions not only demonstrate how rethinking cognitive ability assessment can expand our understanding of the use and relevance of these assessments but also address long-standing challenges–such as the so-called validity/diversity dilemma (Ployhart and Holtz 2008; Scherbaum et al. 2023). These assessments can also better meet the needs of modern workplaces. Each study is discussed in terms of its unique contributions to the literature and its implications for advancing cognitive ability research and practice in organizational contexts.

## 2. Modern Theoretical and Analytical Approaches

Three articles in this Special Issue highlight novel theoretical and analytical approaches to cognitive ability assessment that extend beyond traditional *g*-centric models, offering fresh perspectives on how cognitive abilities influence workplace outcomes.

Kato and Scherbaum (2023) investigate ability tilt—an individual’s relative strength in a specific cognitive ability over another—and its relationship to job performance. Analyzing data from the General Aptitude Test Battery, they found that ability tilt can predict job performance beyond general intelligence (*g*) and specific cognitive abilities. Notably, the impact of ability tilt varies depending on whether the tilt reflects the cognitive demands of the job; for instance, a verbal tilt may benefit performance in roles requiring strong verbal ability but could be disadvantageous in quantitatively oriented positions. These findings suggest that considering ability tilt can enhance personnel selection by aligning individuals’ cognitive profiles with job requirements.

Conte and Harmata (2023) extend the notion of cognitive ability profiles beyond ability tilt by examining more complex profiles of specific abilities. Utilizing latent profile analysis, the researchers identified five distinct cognitive profiles based on individuals’ strengths and weaknesses across six dimensions measured using the Armed Services Vocational Aptitude Battery. The findings reveal that people with similar general intelligence scores could exhibit different patterns of cognitive abilities, underscoring the complexity of cognitive functioning. Additionally, job performance criteria varied significantly across the five cognitive profiles. This work suggests the importance of considering cognitive profiles in occupational settings to better align tasks with individuals’ unique cognitive strengths.

Stamate et al. (2024) consider the implications of cognitive ability for personality structure and expression. Specifically, the authors test the differentiation of personality using the cognitive ability hypothesis, which suggests that personality is more differentiated at higher levels of cognitive ability. By analyzing data from a large sample of job candidates, this hypothesis was supported through the emergence of a sixth factor of personality (beyond the traditional “Big Five”) for individuals with high cognitive ability. These findings have implications for personnel selection and assessment practices, highlighting the importance of considering cognitive ability and personality profiles in tandem.

## 3. Novel Measurement Methods

Three additional articles explore new approaches to cognitive ability measurement, illustrating the breadth of innovation in assessment research and design.

Ohlms and Melchers (2025) investigated how test takers respond to gamified cognitive ability tests across different selection contexts. They compared perceptions in a high-stakes setting, using real applicants for an apprenticeship or dual-study program, with perceptions in a low-stakes, research-simulated context. Interestingly, real applicants (i.e., those in the high-stakes setting) reported more positive reactions to the gamified format than those under the low-stakes condition. Their work highlights the context-dependent nature of applicant reactions and highlights the need to consider the setting when evaluating the effectiveness of gamified assessments.

Bipp et al. (2024) conducted a meta-analysis to examine the construct validity of game-related assessments (GRAs). The authors assessed the relationship between GRAs and traditional cognitive ability tests, noting an overall corrected correlation of *r* = 0.45. Despite observing moderate to strong correlations, the authors shared that GRAs may also tap into traits like engagement, persistence, and gaming familiarity, which are not typically assessed in traditional cognitive ability tests. As such, the authors illustrate that the two should not be treated as equivalent assessments. Their study offers a critical and balanced evaluation of a growing practice in workplace assessments, helping practitioners and researchers better understand both the potential and limitations of GRAs.

Finally, Wang et al.’s (2023) study, using the principles of psychometric AI, presents an innovative use of eye-tracking and machine learning to measure emotional intelligence (EI). Of note, they tested multiple machine learning models and found that certain models could accurately classify individuals with high or low EI based on as little as 2 to 5 s of eye-tracking data, with specific features (e.g., the average duration of blinks, fixation counts, and pupil size) being especially predictive. Their work demonstrates the feasibility of a real-time and non-invasive EI assessment that is less biased and fakable.

## 4. Application of Modern Measures

The final study, conducted by Goldstein et al. (2023), demonstrates the application of a modern cognitive ability test to predict performance in public safety occupations with much lower score differences than are typically seen with traditional cognitive ability tests. This study demonstrates how updated measures can help address the documented trade-off between predictive validity and diversity in high-stakes testing.

## 5. Conclusions

The aim of this Special Issue was to bring together examples of innovation in the conceptualization and measurement of cognitive ability in the modern world of work. Collectively, these studies offer insights into the range of innovations that are possible using modern theories of cognitive ability, modern analytical approaches, and novel measurement methods.
